# THE ROLE OF HOUSING IN LONG-TERM NURSING HOME UTILIZATION IN THE UNITED STATES: AN INTEGRATIVE REVIEW

**DOI:** 10.1093/geroni/igae098.3904

**Published:** 2024-12-31

**Authors:** Marissa Bergh, Jasmine Travers

**Affiliations:** New York University, Long Island City, New York, United States; New York University, New York, New York, United States

## Abstract

Given the growing housing crisis, researchers increasingly point to housing as a possible risk factor for nursing home (NH) utilization, yet the exact pathways from housing to NH utilization are unclear. The purpose of this integrative review was to develop an understanding of how housing acts as a mechanism that impacts NH utilization in the United States. Five databases were searched in April 2024 and 16 total articles were included. This review highlights four pathways for this relationship: 1) Financial Asset vs. Burden, represents how some leverage home equity to help finance necessary NH placements, while for others the cost-burden of housing may lead to early nursing home placements; 2) Access to Affordable Housing, represents how the supply of affordable housing impacts the ability of NH residents to return to community living; 3) (Mis)match Between Needs and Housing, represents how functional needs rapidly evolve, leading to housing transitions and NH placements; and 4) Compounding Systemic Inequities, represents how systemic inequities in access to affordable, quality housing lead to disparities in NH placement for socio-economically marginalized older adults. The results from this review provide evidence of the relationship between housing and NH utilization across these four pathways; however, due to weak quality of evidence, and limited methodological diversity, more research is needed to strengthen this evidence. Clinicians, researchers, and policymakers should still recognize the importance of housing in NH utilization and seek to target policies and interventions towards improving housing conditions for those at risk for NH placement.

